# Exercise Testing and Physical Activity in Dogs: From Health to Heart Disease

**DOI:** 10.3390/ani15223336

**Published:** 2025-11-19

**Authors:** Grégoire Bugeaud, Mário Marcondes-Santos

**Affiliations:** 1 Veterinary Medicine, EUVG—Vasco da Gama University School, Avenida José R. Sousa Fernandes 197, 3020-210 Coimbra, Portugal; 2 CIVG—Vasco da Gama Research Center, Avenida José R. Sousa Fernandes 197, 3020-210 Coimbra, Portugal; mariomarcondessantos@gmail.com; 3 School of Agriculture, Santarém Polytechnic University, Quinta do Galinheiro-S. Pedro, 2001-904 Santarém, Portugal

**Keywords:** canine, heart failure, ergometric test, exercise training, physical exercise, prognostic evaluation, quality of life, veterinary cardiology

## Abstract

The utility of physical exercise in congestive heart failure is not yet well known in the field of veterinary cardiology, despite many studies already published, unlike in human medicine where the benefits and safety of exercise training have been widely proven and included in recommendations and consensus. Several studies have been conducted since the end of the 20th century, evaluating the usefulness of physical exercise in the diagnosis, prognosis, as well as the treatment of congestive heart failure in dogs. The information from these studies has been compiled in this work to conduct a literature review and propose a work base, information, and protocols to develop knowledge about the effect of exercise training on congestive heart failure for future clinical research in dogs. Two major types of exercise tests have been published: the 6 min walk test, easy to implement and seemingly better at reflecting the daily physical capacities of cardiac dogs, and treadmill tests, such as the ergometric test or incremental tests, which, combined with the measurement of plasma concentration of NT-proBNP (N-terminal pro B-type natriuretic peptide), are promising for prognostic evaluation and monitoring of conventional drug therapy. An exercise training program does not reverse the damage caused by congestive heart failure but can help delay and slow the progression of the disease, essentially having effects on heart rate and sympathetic modulation of cardiac activity and preserving cardiac function. Additionally, an improvement in the functional class of heart failure and quality of life due to physical exercise has been observed, a key point for owners. Even though there are risks associated with this complementary therapy (syncope or risk of exacerbating symptoms of cardiac pathologies), the risk–benefit balance seems to clearly favor the use of exercise when used in a controlled manner in stable patients. Evidence of the utility of physical exercise as a testing method or as a complementary treatment has been gathered in this review. However, to further develop the clinical practice of exercise, additional studies need to be conducted to develop standardized testing methods, clarify the impact of exercise training programs on all classes of heart failure, assess the risks, and analyze the long-term effects on canine species.

## Simple Summary

Physical exercise is a well-established supportive therapy in human cardiology, but its use in veterinary cardiology, particularly in dogs with congestive heart failure, remains underexplored. This review compiles existing veterinary studies to provide a basis for the clinical use of exercise in diagnosis, for prognosis, and as a complementary treatment (exercise training). Two main exercise tests have been evaluated: the 6 min walk test, which offers a simple and practical way to assess functional capacity, and treadmill-based protocols, including stepwise or workload tests, which can be paired with biomarkers to monitor therapeutic responses. While exercise training does not reverse myocardial damage, it appears to slow disease progression by modulating sympathetic activity, preserving cardiac function, and improving functional status and quality of life in affected dogs. Improvements in clinical signs and activity tolerance have been reported, which are meaningful outcomes for both clinicians and owners. Although adverse events such as syncope or worsening of clinical signs can occur, especially in advanced cases, the overall risk–benefit profile supports the careful integration of exercise training into case management. This review emphasizes the clinical potential of structured physical activity and underlines the need for further research to standardize protocols and evaluate long-term effects in canine heart failure.

## 1. Introduction

Congestive heart failure (CHF) is considered a pathophysiological condition that occurs when the heart is unable to meet the metabolic demands of tissues and organs or can only do so with excessively elevated filling pressures [1]. This condition is characterized by the activation of the neurohormonal system and its components (angiotensin II, norepinephrine, and vasopressin) [2,3]. In dogs, CHF can be classified into several subtypes depending on the underlying disease: myocardial failure, volume overload, valvular insufficiency, preload deficit, or diastolic dysfunction [1].

The most common heart diseases in dogs are myxomatous mitral valve disease (MMVD), primary arrhythmias, congenital disorders (such as pulmonic stenosis, patent ductus arteriosus, and aortic stenosis), and dilated cardiomyopathy [3,4,5]. Among these, chronic degenerative mitral valve disease, pulmonic stenosis, aortic stenosis, and dilated cardiomyopathy are the conditions most frequently progressing to CHF [1]. However, other less clinically prevalent diseases may also lead to CHF [1].

In humans, the addition of regular and chronic controlled programs of exercise training to conventional pharmacological treatment has been shown to improve the clinical condition and quality of life of patients with CHF of various etiologies [6,7]. Exercise training can even increase patient survival time and reduce hospitalizations [8], as well as improve exercise intolerance [9]. Early initiation of physical activity in the postoperative period in stable patients has demonstrated beneficial effects and does not compromise cardiac repair [10].

Exercise is also routinely used in humans as a diagnostic tool for CHF [11] and has proven to be even more reliable than the New York Heart Association (NYHA) classification system [12]. Several protocols incorporate physical exercise as a complementary test to improve the clinical evaluation of cardiovascular response to physical effort [13]. The main exercise tests used for more accurate diagnostic and prognostic assessment of CHF progression can be classified as: maximal effort tests, submaximal effort tests, and endurance tests [14].

In dogs, the use of physical exercise—specifically submaximal treadmill exercise—as a diagnostic aid is feasible and provides valuable information regarding the patient’s response to treatment [15]. The six-minute walk test (6-MWT) can also be used as a diagnostic tool for CHF, offering a simpler alternative to treadmill-based tests [16].

Controlled sub-lactate exercise (that is, aerobic exercise) in dogs with chronic mitral valve disease may also improve both the quality of life and certain clinical parameters, such as heart rate (HR) and functional class of heart failure [17,18].

Since the first studies conducted in the late 20th century, further research has expanded our understanding of the effects of physical exercise on CHF and its potential applications.

The aim of this narrative review is to discuss the main published studies on physical exercise in dogs, both for diagnostic and prognostic assessment and as a therapeutic strategy (exercise training) to slow the progression of CHF, thereby contributing to a better understanding of this highly prevalent condition in routine veterinary practice.

## 2. Materials and Methods

This narrative review was conducted in accordance with the guidelines for narrative reviews as recommended by the journal Animals. The objective was to identify, analyze, and summarize the current scientific evidence on the use of physical exercise as a diagnostic, prognostic, and therapeutic tool in the management of canine congestive heart failure.

A comprehensive literature search was carried out using the following electronic databases: PubMed, ScienceDirect, and Google Scholar. Articles published from 1990 to September 2025 were considered. The search was performed using the following keywords in various combinations: canine congestive heart failure, dogs, physical exercise, exercise testing, exercise training, treadmill test, 6-min walk test, cardiac function, and veterinary cardiology.

### 2.1. Inclusion and Exclusion Criteria

Studies were included if they met the following criteria:

- Peer-reviewed articles or theses written in English, Portuguese, French or Spanish.
- Original research, case series, or clinical trials involving dogs with confirmed or suspected CHF or healthy dogs monitored in the purpose of having references for healthy patients.
- Studies were included if they investigated the effects or assessment of physical exercise in dogs with cardiac disease or induced or spontaneous CHF, regardless of disease stage. According to the NYHA classification adapted for veterinary use, class I dogs are considered preclinical, meaning they show cardiac abnormalities without clinical signs of CHF. Studies involving healthy dogs were also included, as reference data from healthy subjects are essential to allow meaningful comparison with data from diseased patients.
- Articles discussing the use of physical exercise for diagnostic, prognostic, or therapeutic purposes.

Exclusion criteria included:

- Studies involving species other than dogs;
- Abstracts, conference proceedings, or unpublished data.

### 2.2. Data Extraction and Analysis

Relevant data were extracted manually from selected articles, including study design, population characteristics, type of exercise, protocol used, outcome measures, and key findings related to diagnostic accuracy, prognostic value, and therapeutic impact. The methodological quality of included studies was considered descriptively but not formally assessed, as this a narrative review.

### 2.3. Article Structure

This article is structured as a narrative review of the selected literature. The synthesis of findings is divided into two main sections, each reflecting a specific clinical application of physical exercise in dogs with CHF. The first part focuses on the use of physical exercise as a testing method, including its diagnostic and prognostic relevance, with particular attention to submaximal tests such as the 6-MWT and treadmill-based protocols. The second part addresses the use of programs of exercise training as a complementary therapy, exploring its physiological benefits, effects on clinical parameters, and its potential role in improving quality of life and slowing disease progression. This division allows for a comprehensive understanding of both the prognostic evaluation and therapeutic effect of exercise in veterinary cardiology. Each section is organized in chronological order, highlighting the evolution of scientific knowledge, and conclude after discussing of current limitations and future research perspectives.

## 3. Results

### 3.1. Diagnostic and Prognostic Use of Exercise Testing in Healthy Dogs and in Dogs with Heart Disease (Including Congestive Heart Failure)

In CHF, dyspnea and fatigue are initially evident following physical exertion. Therefore, exercise testing can assist in the early diagnosis of CHF, before clinical signs of congestion become apparent [16]. Unlike in humans, exercise tests are not routinely used in the cardiac evaluation of dogs [19]. The main barrier to implementing routine exercise testing in veterinary practice is the lack of a standardized and objective testing protocol [19]. Several studies have been conducted to evaluate the feasibility and usefulness of exercise testing in the cardiac assessment of dogs with CHF. Ten studies have been found, matching the inclusion criteria. The main points of each study are detailed bellow.

Treadmill-based tests allow dogs to be subjected to different levels of exercise intensity, defined by varying speeds and/or treadmill inclines. The anaerobic or sub-lactic threshold can be determined by measuring blood lactate levels and is defined as the point at which blood lactate exceeds a reference value (1.0 mmol/L). The study by Kittleson, Johnson, and Pion [20] demonstrated that it is possible to reach the anaerobic or sub-lactic threshold through treadmill exercise in both healthy dogs and those with class IV CHF, experimentally induced through rapid ventricular pacing (as classified by the NYHA system adapted for veterinary use). However, the study also showed that it is not safe to push dogs with class IV CHF beyond this threshold. Additionally, it was found that dogs with surgically induced CHF reach the anaerobic threshold at lower exercise intensities compared to healthy dogs (1 mile per hour at a 16% incline vs. 3 miles per hour at inclines of 16%, 22%, or 26%). The study also highlighted that the main limitation to the use of treadmills in such testing is the dogs’ ability to adapt to the equipment, as some animals may require several hours of training before they are comfortable walking on a treadmill [20].

The 6-MWT involves measuring the distance a dog can walk at its own pace and without distractions over a period of six minutes [16]. This test is performed with the dog on a leash, walking in an area free of obstacles and potential distractions (such as people or other animals), typically a hallway or corridor. Dogs should be acclimated to the environment before the test to ensure that external factors do not influence the results; however, this acclimation process is generally faster than with treadmill tests. The study concluded that dogs with surgically induced CHF through rapid ventricular pacing can be evaluated using the 6-MWT, and that these dogs walk a shorter distance compared to healthy dogs (573 ± 85.5 m for healthy dogs vs. 526 ± 99.4 m for dogs with heart failure) [16].

The submaximal treadmill exercise test appears to be a suitable tool for assessing treatment (pimobendan and furosemide) response in naturally acquired CHF dogs. The protocol described by Ferasin and Marcora [15] can be considered incremental, as it consists of three progressively more challenging stages (increasing speed), with rest intervals between each stage. In contrast to previous reports [20], the animals in this study did not exhibit reluctance to exercise on the treadmill. This improved compliance was likely due to owners being instructed to train their dogs to walk on the treadmill beforehand, thereby facilitating the implementation of the test in clinical practice. According to the study, blood lactate concentration and HR appear to be the most reliable indicators for monitoring exercise intensity. The authors also suggest that the observed increase in serum cardiac troponin I (cTnI) may reflect subclinical myocardial injury induced [15]. However, further research involving a larger sample size is needed to confirm the validity of these findings.

The exercise test described by Marcondes-Santos [17,18] is performed on a treadmill, with continuous electrocardiogram (ECG) monitoring throughout physical activity in a special room of cardiac rehabilitation (with emergency equipment and drugs) to test 36 dogs with naturally acquired chronic mitral valve disease. To carry out this test, an adaptation phase to the treadmill—lasting between 7 and 15 days, with sessions 2 to 3 times per week—is required, along with the presence of the owners during the sessions with dogs in a stable phase of CHF (without pulmonary edema or other signs of cardiac decompensation). Resting HR is measured prior to the test, which is then conducted by increasing the treadmill speed by 1 km/h every 2 min. The test is concluded either after 20 min, or earlier if the animal attempts to stop the exercise or shows transient clinical signs such as tachypnea, panting, pale or cyanotic mucous membranes or arrhythmias. The maximum HR reached during the test is recorded, and based on the resting and maximum HR values, a target working HR is calculated. Blood lactate measurements were performed before and after the tests to confirm the aerobic exercice training. The use of continuous ECG monitoring throughout the procedure enhances the safety of the test. Only one animal out of the 36 included in the study failed to adapt to the treadmill, and none of the animals exhibited clinical signs of cardiac decompensation during testing, further supporting the feasibility of treadmill-based exercise protocols in dogs [17,18].

A different study evaluated the 6-MWT in dogs with myxomatous mitral valve disease naturally acquired, including both preclinical (stage B2) and CHF (stage C2) cases, according to the ACVIM consensus classification [21]. The study emphasized the safety, ease of implementation, and low cost of this test, confirming the findings of Boddy et al. [16] and offering an alternative to treadmill tests. It also confirmed that the distance covered by affected dogs is shorter than that covered by healthy animals and that a healthy geriatric small-breed dog (~9 years, ~10 kg) typically covers approximately 450 m in six minutes [21]. Furthermore, it was demonstrated that the more advanced the stage of the disease (as classified by the ACVIM consensus), the shorter the distance traveled. The study also highlighted several limitations in conducting and ensuring the reliability of the test, such as corridor length (a shorter corridor results in more turns, leading to a reduced distance covered), breed shape and size (breeds with short legs have a less efficient stride pattern), undetected concurrent diseases (neurological and/or orthopedic), and the motivation of the animal and/or the owner [21].

Several studies have evaluated exercise testing in healthy dogs and in dogs with preclinical (stage B1–B2) MMVD. For example, several attempts to conduct standardized exercise tests in dogs on treadmills [22] investigated low and high intensity treadmill tests in healthy Australian Cattle Dogs and Border Collies, and found that only 28% of animals were willing to complete the protocol, suggesting a major feasibility limitation [22] as previously reported in animals with CHF [20]. In dogs with presymptomatic naturally acquired MMVD (class I of the NYHA classification modified for veterinary use), Wall et al. [19] applied a six-stage submaximal treadmill test (three minutes per stage, incremental incline) and measured HR, lactate, acid-base status, and the cardiac biomarkers N-terminal pro-B-type natriuretic peptide (NT-proBNP) and cTnI. They demonstrated that dogs with MMVD had significantly higher baseline NT-proBNP and cTnI than healthy dogs, and exhibited greater post-exercise increases in these biomarkers (for example NT-proBNP increased from 690 to 815 pmol/L in the MMVD group vs. 435 to 523 pmol/L in healthy dogs). They also found that lactate at 3 h post-exercise was significantly higher in the MMVD group compared to healthy controls, whereas pH changes were similar across groups. Although HR responses did not differ significantly between groups, the test proved feasible in a clinical setting and the authors suggest that measuring pre and post-exercise biomarkers is more informative than sampling mid-exercise [19]. These findings support the potential usefulness of standardized submaximal treadmill testing and biomarker assessment to detect early cardiac disease-related exercise intolerance, though the authors note limitations such as differences in breed, age, and lifestyle between groups [19].

The study by Sutayatram et al. [23] confirmed once again that both the ergometric test described by Marcondes-Santos [17,18] and the 6-MWT proposed by Boddy et al. [15] are feasible in terms of safety and technical execution. This study focused on dogs with naturally acquired MMVD at stage B1 of the ACVIM classification, without cardiac remodeling [23]. In these animals the tests appear to be safe and applicable at an early stage of the disease. However, previous results obtained in more advanced stages, in which CHF is already established, showed that the tests or physical training programs should be performed in stable patients (without signs of pulmonary edema or cardiac decompensation) and conducted with appropriate precautions [18,23].

In addition to serving as a diagnostic tool, exercise testing can also be used to monitor the efficacy of cardiac treatments in dogs. In the study by Iwanuk and colleagues [24], asymptomatic dogs classified as ACVIM stage B1—i.e., with preclinical naturally acquired MMVD without cardiomegaly—underwent a standardized submaximal treadmill exercise test. The protocol was performed on the first day of treatment with pimobendan and repeated after 90 and 180 days. Plasma concentrations of cardiac biomarkers, including NT-proBNP and cTnI, were measured immediately before and after each exercise session. The submaximal exercise test was designed to induce moderate and reproducible cardiac wall stress without reaching maximal exertion levels, thereby allowing a controlled physiological assessment of biomarker response. The study showed that dogs receiving pimobendan had significantly lower post-exercise NT-proBNP concentrations at day 180 compared with baseline, and both pre- and post-exercise NT-proBNP values were lower than in the placebo group. No significant differences were found in cTnI concentrations between groups or over time. These findings suggest that pimobendan may reduce myocardial wall stress during physical activity in dogs with preclinical mitral valve disease. Consequently, standardized submaximal exercise testing appears to be a useful and sensitive approach for evaluating therapeutic efficacy and disease progression, supporting the conclusions of Ferasin and Marcora [15,24]. Nevertheless, further validation of such protocols in dogs with more advanced stages of CHF remains warranted [24].

The 6-MWT has been conducted under various conditions, including at different altitudes [25]. This study was carried out in dogs affected by naturally acquired MMVD at stage B2 of the ACVIM classification. The absence of adverse effects demonstrated that the test is safe even for diseased patients at high altitudes (2650 m above sea level). The distance walked by the group of affected animals (356.14 ± 92.13 m) was shorter compared to that of healthy animals. Vargas-Pinto et al. [26] reported a mean distance of 537.4 ± 123.8 m for healthy dogs at high altitude, a value that is relatively low and comparable to the distance measured at sea level (350.04 m) in the study by Agudelo and Schanilec [21] in stage C2 patients. This suggests that high altitude may result in a more pronounced reduction in walking distance [25]. The authors also confirmed that the 6-MWT is easy to perform, low-cost, and generally well tolerated by canine patients, although its results may be limited in the presence of comorbidities (e.g., respiratory conditions). They recommend the use of this test as a tool for monitoring the physical condition of patients over time [25].

### 3.2. Physical Exercise (Training Programs) in Healthy Dogs and Dogs with Heart Disease (Including Congestive Heart Failure)

Physical exercise can also be employed as a complementary therapy in CHF, in conjunction with pharmacological treatments such as digoxin, furosemide, spironolactone, angiotensin-converting enzyme inhibitors, and/or carvedilol [17,18,27]. Initial studies were conducted in dogs used as experimental models of human heart disease, aiming to improve CHF treatment in the human population [28]. More recently, multiple studies have focused on improving the condition in dogs suffering from CHF, both in terms of quality of life and functional classification [18], as well as various clinical parameters in healthy dogs [29]. Twelve articles have met the inclusion criteria. The main points of each study are described in this part.

An initial assessment of the effects of physical exercise training was conducted in dogs with induced CHF, used as an animal model of human CHF. The exercise protocol consisted of one week of submaximal treadmill exercise (4.4 ± 0.3 km/h, one hour in the morning and one hour in the afternoon), performed at the end of the CHF induction phase. Hemodynamic parameters were evaluated in vivo, and the hearts were subsequently collected, prepared, and analyzed. Dogs subjected to the exercise program did not exhibit the classical signs of CHF observed in the control group, such as facial and abdominal wall edema, ascites, labored breathing, and loss of appetite. Compared to sedentary CHF dogs, exercise-trained animals showed attenuated left ventricular remodeling, characterized by lower end-diastolic left ventricular pressure, improved diastolic compliance, and reduced myocardial stiffness. Histological analysis also revealed a slight decrease in myocardial collagen content, indicating partial prevention of fibrosis. Functional assessment demonstrated that physical conditioning preserved global cardiac performance, particularly diastolic function, while maintaining mean aortic pressure, HR, and peak rate of left ventricular pressure rise. Overall, the study concluded that physical exercise training mitigates both structural and functional deterioration in CHF, helping preserve hemodynamic stability and delaying disease progression, although it cannot completely reverse established hemodynamic impairment [28].

Another study using dogs as an animal model focused on the impact of physical exercise training in subjects with surgically induced myocardial ischemia [30]. The training program consisted of five sessions per week for 10 weeks, with progressive increases in intensity, speed, and duration. During these sessions, the dogs ran on a treadmill at 70–80% of their maximum HR. HR and heart rate variability (HRV) were measured and analyzed. The study showed that physical training led to a reduction in HR, an increase in HRV, and a complete suppression of ventricular fibrillation induced by acute myocardial ischemia. These findings suggest that the effects of exercise training are associated with modulation of the exaggerated sympathetic activity observed during the clinical progression of CHF. The training also appeared to restore autonomic cardiac balance and enhance parasympathetic cardiac regulation [30]. These effects imply a reduced risk of sudden cardiac death and clinical signs of CHF. Previous studies conducted by Marcondes-Santos [3] showed that increase in sympathetic activation in heart failure dogs detected by high levels of plasmatic catecholamines (measured after a period of rest, in a calm environment) were associated with poor prognostic and worsening of CHF functional class. A modulation of this activation by parasympathetic cardiac regulation due to exercise training could be the reason of the clinical improvement of these animals [17,18,30].

The effect of physical training was also assessed after induced myocardial infarction [31]. In this study, dogs were again used as models of human cardiac pathologies, even though naturally acquired myocardial infarction is rare in dogs [32], but may progress to CHF [1]. The effects of physical exercise training were assessed by evaluating HR and HRV [31]. The training program followed the same protocol previously used by Billman and Kukielka [30]. This study demonstrated that physical training attenuated the increase in HR and resulted in a smaller decrease in HRV during a subsequent exercise test. These results confirmed an improvement in parasympathetic cardiac modulation, supporting the findings from the earlier study by Billman and Kukielka [30], and suggesting potential benefits for dogs with heart disease by reducing the incidence of adverse rhythm disturbances and decreasing cardiac-related mortality [31].

Physical training programs can also be initiated after an exercise test, which provides a baseline for personalized training [17]. In this protocol, the target HR for aerobic training was calculated based on the maximum HR reached during the ergometric test, and bloodlactate measures before and after the tests, as previously described by Marcondes-Santos [17,18]. Training sessions were conducted twice per week for 20 min each, over a period of three months, at the treadmill speed required to reach the target HR. The first observation was that dogs undergoing the training protocol were able to tolerate progressively higher treadmill speeds, indicating improved physical capacity and endurance [17,18]. A reduction in HR, an improvement in quality of life (assessed via FETCH questionnaire of quality of life), and a clinically relevant improvement in CHF functional class were also reported [17,18,33]. The training protocol also helped stabilize NT-proBNP levels after 6 months of folow-up [18]. However, other evaluated parameters, particularly echocardiographic indices and blood pressure measurements, did not show significant changes [17,18]. Despite some limitations in the study—such as the small number of animals (*n* = 36) and the short duration of the training program—the benefits of aerobic exercise training support the recommendation of light walks lasting 20 to 30 min two to three times per week for stable dogs with CHF. This approach could address one of the main challenges in prescribing physical activity: the need for owners to be available and personally involved in taking their dogs to training sessions with specialized veterinary cardiologists [18].

Valandro and colleagues [34] proposed an exercise training program for dogs affected by naturally acquired MMVD in ACVIM stages B1 and B2. The training was conducted on an outdoor track and consisted of walking at a speed sufficient to achieve a HR between 60% and 80% of the dog’s maximum HR, as determined by 24 h Holter monitoring. Sessions were carried out three times per week for 30 to 50 min (with the session ending either at 50 min or when the dog could no longer maintain the minimum workload). Activity duration, distance covered, average speed, and HR were recorded for each session, and 24 h Holter ECGs were performed at baseline, after four weeks, and after eight weeks of training. No adverse effects were reported, leading to the conclusion that this protocol is safe for dogs with MMVD in stages B1 and B2. The safety of this approach is attributed to maintaining HR between 60% and 80% of the maximum effort level, thereby avoiding ranges associated with adverse effects. This suggests the protocol could potentially be safe even for dogs in more advanced stages, including those with established CHF, but clinically stable. However, studies with these different stages still need to be carried out to confirm this hypothesis. Holter recordings indicated an increase in parasympathetic tone [34]. Given that reduced parasympathetic tone and increased sympathetic activity are key mechanisms in the development of CHF [3,35], the observed increase in parasympathetic tone may contribute to delaying disease progression and onset of CHF, improving survival rates and reducing the risk of sudden cardiac death [3,18,36]. Most owners also reported greater activity levels in their dogs at home following the introduction of the training program, indicating improved quality of life, which supports findings from Marcondes-Santos [18,34].

In addition to evaluating two physical exercise tests, Sutayatram and colleagues [23] assessed a submaximal physical training program for dogs with naturally acquired MMVD in ACVIM stage B1. The training program was conducted twice weekly for eight weeks. Each 20 min session included five minutes of warm-up, a three-minute phase of gradually increasing speed to reach the velocity that caused exhaustion in the previous session or test, a main phase during which the speed was adjusted according to each dog, and a four-minute cool-down. Hematological, blood biochemical, echocardiographic, and electrocardiographic parameters were measured at baseline, after four weeks, and after eight weeks of training. No adverse effects were observed during the training, demonstrating the safety of the program in dogs with MMVD at stage B1. Additionally, no reluctance toward the treadmill was noted, which the authors attributed to the encouraging gestures and verbal reinforcement provided by the team. Although the protocol showed improvements in the physical condition of the trained dogs—potentially contributing to a better quality of life—it did not result in statistically significant changes in the measured parameters. This could be due to study limitations such as the training duration, intensity, or sample size. Another possibility is the subclinical status of the disease in the study population, as the dogs presented only mild cardiovascular changes and most evaluated parameters remained within normal reference ranges [23].

Another study investigated the effect of sub-lactate threshold training on HR and several cardiac biomarkers: NT-proBNP, cTnI, and the myocardial band of creatine kinase (CK-MB) [29]. The study was conducted in young, healthy dogs. Following a 10-day adaptation period to treadmill exercise, during which dogs were encouraged by the research team, the training program consisted of 30 min sessions—comprising five minutes of warm-up, 20 min of activity, and five minutes of cool-down—three times per week over eight weeks. During the first four weeks, the activity phase was performed at 70% of the speed corresponding to the lactate threshold, as determined through an incremental exercise test prior to training; during the last four weeks, this intensity was increased to 80%. Warm-up and cool-down speeds were set at 50% of this threshold speed. The study demonstrated an improvement in cardiac functional capacity and overall physical fitness in the dogs. The cardiac biomarkers increased transiently post-exercise but returned to baseline within 48 to 72 h, and no persistent elevations were observed. These fluctuations suggest that the training regimen did not cause myocardial damage, indicating a high level of safety—an essential consideration for prescribing sub-lactate exercise programs in animals with cardiac disease [29].

Several exercise program models were initially developed for humans before being adapted for dogs, such as the FITT-VP principle (Frequency, Intensity, Time, Type—Volume, Progression), which Lee and collaborators [36] attempted to apply in healthy dogs. This principle relies on the combined modulation of parameters to tailor the program to the patient and ensure progressive improvement, implemented here through programs composed of 12 protocols, each divided into several stages. A continuous exercise program (i.e., without rest intervals) and an interval training program (i.e., with rest or active recovery intervals) were tested over four weeks with three training sessions per week. Both programs were conducted without any adverse effects, and hematological parameters remained unchanged, demonstrating the safety of these approaches. The study also reported a temporary increase in serum creatine kinase, suggesting minimal musculoskeletal damage resulting from exercise. HRV was correlated with exercise intensity in all interval protocols and only in the more intense continuous protocols [36].

Another study investigated the effects of sub-lactate threshold training on electrocardiographic parameters in healthy dogs [37]. Animals first underwent an incremental exercise test to determine the lactate threshold, which was then used for the individual prescription of training intensity. The exercise program lasted eight weeks, with three sessions per week, following a 10-day adaptation period [37]. Each session was divided into three phases, following the same structure as described by Cerqueira et al. [29]. No adverse events were observed during the study, although one of the 20 dogs failed to adapt to the treadmill used for training. The study demonstrated an increase in HRV and a decrease in mean HR—findings consistent with those of Billman and Kukielka [30], who observed similar changes in dogs with experimentally induced myocardial ischemia. Increased HRV reflects enhanced parasympathetic tone and reduced sympathetic regulation, indicating a restoration of autonomic balance, which is critical for preventing and slowing the progression of CHF. The simultaneous reduction in HR reinforces this observation, as reduced resting HR is considered a marker of improvement in CHF. However, these findings in healthy dogs need validation in animals with cardiac disease [37].

A further study by Restan and collaborators [38] evaluated the influence of a physical training program on echocardiographic parameters in healthy dogs. The animals first underwent an incremental exercise test to determine the aerobic threshold and corresponding speed, which was then used to design an individualized training protocol, based on previous methodologies described by the same research group [29,37]. This study demonstrated that the training program improved both diastolic and systolic cardiac function through physiological adaptations, including ventricular dilation, enhanced early diastolic relaxation, and improved radial systolic mechanics of the left ventricle. Since these functions are commonly impaired in cardiac pathologies and contribute to the development of CHF, the results are promising for the use of exercise as an adjunctive therapy alongside pharmacological treatment. Nevertheless, confirmation in dogs with cardiac disease is still required. It is worth noting that one of the 18 dogs developed mitral valve regurgitation at the end of the training program, which resolved spontaneously after a 10-week rest period. This adverse effect has been described in humans, horses, and trained dogs, and is considered physiological—stemming from cardiac remodeling due to training rather than pathology—and was reversible after exercise cessation. However, further investigation is necessary to determine the incidence of this effect, which appears to be associated with high-intensity training and could potentially exacerbate certain cardiac conditions. Additionally, the timeframe required for reversal should be evaluated, especially in the context of using physical exercise as a complementary therapy in dogs with CHF [38].

## 4. Discussion

### 4.1. Diagnostic and Prognostic—Use of Exercise Testing in Healthy Dogs and in Dogs with Heart Disease (Including Congestive Heart Failure)

These studies examined two types of exercise tests: treadmill-based tests, such as the one described by Kittleson, Johnson, and Pion [20], and the 6-MWT, as characterized by Boddy and colleagues [16]. Treadmill tests can be further categorized into several subtypes, depending on the specific protocol employed, such as the ergometric test [17] or incremental protocols like the one described by Wall et al. [19]. All these types of tests are summarized in Table 1.

Currently, the main challenges in conducting exercise tests for cardiac evaluation include: the lack of reference data and the lack of a gold-standard method [19]; variations in body size due to breed differences, which affect test outcomes [19,21]; and issues related to the cooperation of both animals and their owners, including the degree of owner involvement [15,17,23]. Each type of test presents various advantages and disadvantages (see Table 2), but both approaches show promises for assessing cardiac function. However, the 6-MWT appears to be more effective in predicting daily physical capabilities compared to treadmill-based tests [23].

Exercise testing is not yet included in current guidelines for the diagnosis of cardiac diseases and CHF [39], but it could already serve a valuable role in classifying affected animals and monitoring treatment response. Once the challenges to its clinical and systematic implementation are addressed, such testing should be incorporated into the routine diagnostic and discussed in consensus guidelines of heart diseases tools used by clinicians.

In humans, the increase in NT-proBNP after exercise testing has proven crucial in the prognosis and classification of patients with mitral regurgitation, allowing the identification of asymptomatic patients who will develop severe cardiac symptoms (CHF or acute pulmonary edema). Patients at risk of developing severe cardiac symptoms and those with a much lower risk have similar NT-proBNP levels at rest, but at-risk patients show a more significant increase after exercise compared to other patients [40]. If these results are confirmed in dogs, they could provide highly relevant information, highlighting the importance of performing exercise tests in dogs diagnosed with CHF.

### 4.2. Physical Exercise Training in Healthy Dogs and Dogs with Heart Disease (Including Congestive Heart Failure)

In humans, the benefits and safety of physical training as a complementary therapy alongside medication have been recognized and implemented in CHF guidelines for over 10 years [41]. The benefits and safety have even been proven in the long term through randomized clinical trials [42]. Currently, for dogs, physical exercise training program is neither mentioned in the guidelines of canine heart diseases nor in the recent literature reviews as a complementary therapy [27,39], despite evidence of its usefulness in the treatment of heart diseases, particularly CHF (see Table 3). Although these findings are limited due to the small number of animals involved in each study and the small number of studies, evidence of its benefit has been clearly demonstrated, especially in the studies of Marcondes-Santos et al. [18], Valandro et al. [34], and Sutayatram et al. [23]. The biggest challenge in obtaining more reliable data is that studies are complex to conduct in veterinary medicine due to their long duration, high costs, and potential patient dropouts during the study. Randomized clinical trials have proven to be a good alternative [27]. Clear discussion based in previous studies with physical exercise training programs should be conducted in the next new consensus guidelines for treatment and diagnosis of different canine heart diseases, with a specific topic just to discuss physical exercise, similar to humans guidelines.

Physical exercise training could be used across all functional classes (modified NYHA classification for veterinary) or ACVIM functional classification for canine CHF, providing benefits to the patient. However, more precautions must be taken as CHF and the underlying disease progress, with patients becoming more sensitive to the adverse effects of physical exercise in more advanced functional classes of CHF. Physical training is contraindicated in patients with clinical signs of congestion or with pulmonary edema. These patients should first be stabilized through medication before being subjected to physical training. Exercise-induced seizures are exceptionally rare [43], but could be a severe adverse effect, often accompanied by additional cardiovascular symptoms or the worsening of pre-existing symptoms, as described by Restan et al. [38]. In humans, the risk–benefit balance clearly favors the use of physical exercise as a complementary therapy, as long as it is performed carefully in hemodynamically stable patients or animal models [31,44,45]. Although not as extensively proven in dogs, the limited number of reported adverse effects in the studies reviewed in this work suggests that the risk–benefit balance in dogs also supports the use of physical exercise training as a complementary therapy in stable patients, in a controlled environment, and new randomized studies with a large number of animals should be conducted to improve knowledge in this new area of the veterinary cardiology.

Some limitations in the use of physical training in dogs include maximizing safety, performance, and acceptance of training by the dogs [37]. Therefore, some new methods are currently being developed, such as a visual exhaustion scale [46], which could improve the safety of treadmill training; standardized physical training protocols that have been tested multiple times, such as those described by Cerqueira et al. [29] and Restan et al. [37,38], which would ensure greater performance in animals; or a standardized method for habituating dogs to treadmills, regardless of speed or incline [47], which would allow for better acceptance by the animals. However, all these new procedures still need to be tested in animals with cardiac pathologies and CHF.

Regarding safety, almost all studies agree on using a heart rate between 60% and 80% of the maximum HR, which would prevent the appearance of most potential adverse effects, as it does not reach heart rate values associated with adverse events. To further ensure safety, Restan et al. [38] recommend determining the speed corresponding to the lactate threshold through an incremental exercise test and then performing the program at a speed between 70% and 80% of the lactate threshold speed, as it has been shown in horses that this reference value is more reliable and safer than the maximum heart rate or maximum oxygen consumption [48]. Also, to increase safety, it is important to monitor heart rate during exercise sessions, regardless of the CHF class, since heart rate changes may reflect pain, stress, or adverse effects [49]. In this regard, the use of a heart rate monitor seems to be the best compromise between safety, reliability, cost, and ease of implementation in clinical practice [17,18,49]. However, it has also been observed that low-intensity training (less than 50% of maximum oxygen consumption) may not provide cardiovascular benefits [37]. The aerobic exercise training suggested by Marcondes-Santos [17,18] in a controlled physical training program could be safe and promote clinical improvements, using the monitoring of maximum heart rate associated with blood lactate measurements in a previous ergometric test. Then, the following trainings could be monitored by the target heart rate calculated in the ergometric test. After some time, new ergometric tests should be performed to achieve higher heart rate values due to the better physical conditioning of the patients [17,18]. To conduct this protocol, a veterinary heart rehabilitation room was designed with environmental temperature control, oxygen therapy equipment, emergency drugs, and a defibrillator. A suitable dog treadmill with digital speed control was used. Cardiac monitoring was performed using ergometric equipment connected to a microcomputer (Cardiobyt, São Paulo, Brazil) and an HR monitor (Polar Electro, Lake Success, NY; USA). The training heart rate (THR) was calculated for aerobic exercise using the reserve HR. The reserve HR was calculated as the difference between the maximum heart rate (HHR) and the heart rate at rest (RESTHR) during the effort test. The THR was calculated as 50% to 70% of the reserve HR in addition to the RESTHR according to the following formula: THR = (HHR − RESTHR) × 50–70% + RESTHR. To confirm the aerobic training, tests using blood lactate were used [17,18].

**Figure 1:**
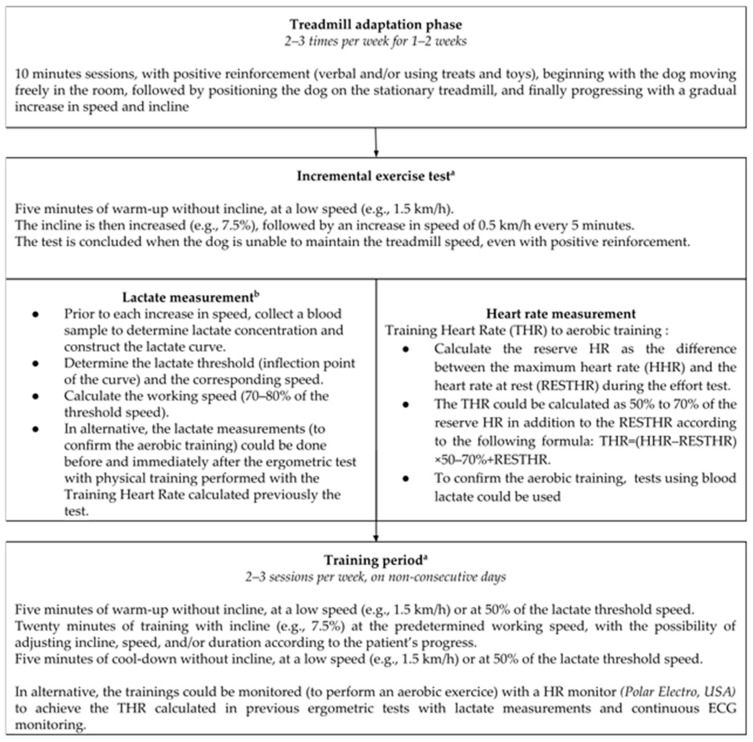
Suggestion of Standardized Physical Exercise Program Training for Research in Cardiac Rehabilitation of Dogs with Congestive Heart Failure (Adapted from on Marcondes-Santos [17,18], Cerqueira et al. [29], Restan et al. [37,38], and Stigall et al. [47]). a: “Phase that should be performed with heart rate monitoring (Holter or heart rate monitor).” b: “The lactate method appears to be more reliable and safer and can be combined with heart rate monitoring for supplementary safety.”

All this information allows the drafting of a pattern for the implementation of a physical exercise training program, which could be applied for research about the effects of clinical exercise training (Figure 1).

Although different physical exercise training programs may not be able to reverse the damage caused by heart diseases and CHF, it has been proven that they can slow the progression of CHF or delay the development of the disease. Therefore, it is crucial to implement these programs across all functional classes, as long as the animals are compensated for signs of congestion and pulmonary edema and are free from dyspnea. Actually, it might be even more important to subject animals in functional class I (modified NYHA classification for veterinary use) to physical exercise training programs, as they are at lower risk of developing adverse effects and this could prolong the period without signs of CHF. This would imply that animals in compensated stages B1, B2, and C (according to the ACVIM staging) would be ideal candidates for this complementary therapy. Even more importantly, physical exercise training improves the quality of life of animals [18,34], which is one of the most important aspects indicated by owners. A low quality of life is one of the major factors driving the decision for euthanasia [50]. Thus, in addition to improving the clinical status of the animal and slowing the disease progression, this improvement in quality of life is one of the most relevant points to the use of physical exercise training in the treatment of CHF and cardiac diseases.

It is also important to recognize that dogs affected by CHF are typically elderly animals, which may have concomitant diseases (such as osteoarthritis) that can influence their physical abilities [23]. These potential comorbidities should be considered when prescribing physical exercise training programs, and in case of doubt, the precautionary principle should be applied, prescribing less intense exercise, which will bring fewer cardiovascular benefits but will not jeopardize the patient’s health [23].

## 5. Conclusions

Physical exercise is routinely used in human cardiology, both for the diagnosis and classification of patients with congestive heart failure, and as a supportive tool in pharmacological and surgical therapies. This contrasts with current practices in veterinary cardiology, where its application remains limited.

Exercise tests, such as incremental treadmill tests or the six-minute walk test, appear promising for the diagnosis and classification of canine patients with CHF. These tests may also provide prognostic value—particularly through the measurement of NT-proBNP response to exercise—and represent a potentially effective method for assessing response to medical treatment.

Regarding structured exercise training programs, current evidence suggests that they improve clinical status, functional class, and quality of life in dogs with CHF, while also slowing the progression of cardiac disease and CHF by preserving cardiac structure and function.

The findings discussed in this work support the implementation of controlled physical activity (e.g., leash walking with owners or treadmill exercise) in dogs with compensated CHF, with the aim of enhancing quality of life and mitigating disease progression. Despite the existence of some published studies in veterinary medicine, there is a need for further randomized clinical trials involving larger cohorts and covering a wider range of canine cardiac pathologies to use these protocols in the routine of the veterinary clinic in near future. This would enable a deeper understanding of the effects of physical exercise in canine CHF patients, ultimately improving diagnosis and treatment strategies.

## Figures and Tables

**Table 1:** Summary of Published Studies on the Use of Physical Exercise for Diagnostic and Prognostic Assessment in Healthy Dogs, Dogs with Preclinical Heart Disease, and Dogs with Congestive Heart Failure.

Type of Evaluated Test [Reference]	Population Involved	Main Conclusions
Sub-lactic incremental treadmill test [20]	12 healthy dogs 12 dogs with iatrogenic class IV CHF (modified NYHA classification for veterinary use)	Allows distinction between healthy dogs and dogs with class IV CHF (which reach the lactate threshold at a lower level of difficulty) Reluctance to walk on the treadmill in some dogs
6-MWT [16]	16 healthy dogs 16 (the same) dogs with iatrogenic CHF	Dogs with CHF cover a shorter distance compared to healthy dogs in six minutes
Sub-lactic incremental treadmill test [15]	7 dogs with naturally acquired CHF (class II or III of the modified NYHA classification for veterinary use)	Lactate and heart rate appear to be the best markers for reflecting exercise levels The exercise test seems to provide useful information for evaluating the individual treatment response If the owner stimulates and trains the dog on the treadmill, there does not appear to be any reluctance in performing the test
Ergometric test [17,18]	36 dogs with naturally acquired CHF due to MMVD (all classes of the modified NYHA classification for veterinary use)	Test with high safety and no reluctance from the dogs, provided they are accustomed to the environment and the owners remain in front of the dog during the test. Additional support with electrocardiography and measurements of blood lactate before and after previous ergometric tests ensure a safe study to design programs of aerobic exercise training
6-MWT [21]	14 healthy dogs 24 dogs with MMVD naturally acquired in B1 or B2 stage of ACVIM classification	It demonstrated that the test is safe, cheap, easy to perform, and well-accepted by the animals It confirmed that the heart disease animals cover a shorter distance and determined that the distance is even more reduced as the disease progresses
Sub-lactic incremental and continuous treadmill test [22]	39 healthy dogs	Issue of reluctance from animals in performing the test (24 dogs were unable to complete both tests) There were no statistical differences between breeds for the evaluated parameters
Sub-lactic incremental treadmill test [19]	12 healthy dogs 12 dogs with MMVD naturally acquired in B1 or B2 stage of ACVIM classification	It showed a more significant increase in NT-proBNP and cTnI induced by exercise in heart diseased dogs compared to healthy dogs
6-MWT and ergometric test [23]	6 dogs with MMVD naturally acquired in B1 stage of ACVIM classification	Feasible and safe tests With positive reinforcement, there was no reluctance from the animals to walk on the treadmill
Ergometric test [24]	21 dogs with MMVD naturally acquired in B1 stage of ACVIM classification	It showed that physical exercise can be a good method for assessing treatment response
6-MWT [25]	7 dogs with MMVD naturally acquired in B2 stage of ACVIM classification	Influence of high altitude on the distance covered by the dogs (reduced distance), but the test remained safe even at high altitude

**Table 3:** Summary of the Main Published Studies on Physical Exercise Training in Healthy Dogs, Dogs with Preclinical Heart Disease, and Dogs with Congestive Heart Failure.

Authors [Reference]	Population Involved Healthy/Sick		Main Conclusions
Todaka et al. [28]	None	12 dogs with induced CHF, class IV of NYHA (6 subjected to the exercise training program and 6 not subjected)	Physical training is beneficial for cases of CHF, preserving cardiac function, and should be initiated as early as possible in the development of CHF to preserve cardiac function
Billman and Kukielka [30]	None	36 dogs with induced CHF (17 subjected to the exercise training program and 19 not subjected)	The effect of physical training is related to a modulation of sympathetic activity, it can restore cardiac autonomic balance and increase parasympathetic cardiac regulation
Kukielka, Seals and Billman [31]	None	16 dogs with induced CHF (9 subjected to the exercise training program and 7 not subjected)	An improvement in parasympathetic cardiac modulation was observed
Marcondes-Santos [17], Marcondes-Santos et al. [18]	10 healthy dogs	36 dogs with naturally acquired CHF, with all functional classes of the modified NYHA classification for veterinary use represented (23 subjected to the exercise training program and 13 not subjected)	An improvement in quality of life, heart rate, and functional class was observed
Valandro et al. [34]	None	20 dogs with naturally acquired MMVD in stages B1 and B2 of ACVIM classification (11 subjected to the exercise training program and 9 not subjected)	Increase in parasympathetic activity and improvement in quality of life
Sutayatram et al. [23]	None	6 dogs with naturally acquired MMVD (stage B1 of the ACVIM classification)	It demonstrated an improvement in physical condition, which could be responsible for an enhancement in quality of life
Cerqueira et al. [29]	18 healthy dogs	None	Improvement in cardiac functional capacity and physical condition of the dogs Transient increase in cardiac marker values, indicating the absence of myocardial damage
Lee et al. [36]	4 healthy dogs	None	Both continuous and interval FITT-VP protocols are safe Low-intensity protocols do not seem to provide benefits to the cardiac system
Restan et al. [37]	20 healthy dogs	None	Increase in parasympathetic tone and decrease in sympathetic activity Decrease in resting heart rate
Restan et al. [38]	18 healthy dogs	None	Improvement in cardiac diastolic and systolic function due to physiological adaptations

**Table 2:** Comparison of the Key Advantages and Disadvantages of Treadmill Tests and the Six-Minute Walk Test.

	Advantages	Disadvantages
Treadmill tests	Different levels of difficulty [20] Higher safety, especially the ergometric test [17,18] Good reliability [19,20]	Significant adaptation time required to avoid reluctance from dogs to walk on the treadmill [15] Cost and complexity of protocols [19] Need to define markers for evaluating cardiac function [15,17]
6-MWT	Low cost, easy to perform, and easy to implement [21] Short habituation time [16] Safe because it is performed at the patient’s pace [21,23]	Risk of animal distraction during the test [16] Limitations due to corridor size and breed size and shape [21] Performed outside the hospital environment, which makes any medical intervention during the test difficult, if necessary, as suggested by other controlled tests [17,18] Influence of altitude and possibly other environmental parameters on the distance covered [25]

## Data Availability

No new data were created or analyzed in this study.

## Abbreviations

The following abbreviations are used in this manuscript:

ACVIM	American College of Veterinary Internal Medicine
CK-MB	Creatine Kinase Myocardial Band
CHF	Congestive Heart Failure
cTnI	Cardiac Troponin I
ECG	Electrocardiogram
FITT-VP	Frequency, Intensity, Time, Type—Volume, Progression
HHR	Maximum Heart Rate
HR	Heart Rate
HRV	Heart Rate Variability
MMVD	Myxomatous Mitral Valve Disease
NT-proBNP	N-terminal pro-B-Type natriuretic peptide
NYHA	New York Heart Association
RESTHR	Heart Rate at Rest
THR	Training Heart Rate
